# Deciphering Electrolyte Dominated Na^+^ Storage Mechanisms in Hard Carbon Anodes for Sodium‐Ion Batteries

**DOI:** 10.1002/advs.202305414

**Published:** 2023-10-24

**Authors:** Guiyu Liu, Zhiqiang Wang, Huimin Yuan, Chunliu Yan, Rui Hao, Fangchang Zhang, Wen Luo, Hongzhi Wang, Yulin Cao, Shuai Gu, Chun Zeng, Yingzhi Li, Zhenyu Wang, Ning Qin, Guangfu Luo, Zhouguang Lu

**Affiliations:** ^1^ Department of Materials Science and Engineering Shenzhen Key Laboratory of Interfacial Science and Engineering of Materials Southern University of Science and Technology Shenzhen 518055 China; ^2^ Guangdong Provincial Key Laboratory of Computational Science and Material Design Southern University of Science and Technology Shenzhen 518055 China

**Keywords:** electrolyte, electron paramagnetic resonance, hard carbon, in situ Raman, sodium storage mechanism

## Abstract

Although hard carbon (HC) demonstrates superior initial Coulombic efficiency, cycling durability, and rate capability in ether‐based electrolytes compared to ester‐based electrolytes for sodium‐ion batteries (SIBs), the underlying mechanisms responsible for these disparities remain largely unexplored. Herein, ex situ electron paramagnetic resonance (EPR) spectra and in situ Raman spectroscopy are combined to investigate the Na storage mechanism of HC under different electrolytes. Through deconvolving the EPR signals of Na in HC, quasi‐metallic‐Na is successfully differentiated from adsorbed‐Na. By monitoring the evolution of different Na species during the charging/discharging process, it is found that the initial adsorbed‐Na in HC with ether‐based electrolytes can be effectively transformed into intercalated‐Na in the plateau region. However, this transformation is obstructed in ester‐based electrolytes, leading to the predominant storage of Na in HC as adsorbed‐Na and pore‐filled‐Na. Furthermore, the intercalated‐Na in HC within the ether‐based electrolytes contributes to the formation of a uniform, dense, and stable solid–electrolyte interphase (SEI) film and eventually enhances the electrochemical performance of HC. This work successfully deciphers the electrolyte‐dominated Na^+^ storage mechanisms in HC and provides fundamental insights into the industrialization of HC in SIBs.

## Graphical abstract

Hard carbon (HC) is a promising anode for SIBs. But the understanding storage mechanism in HC under different electrolytes remain largely unexplored. To address this gap, ex situ EPR spectra and in situ Raman spectroscopy are combined to investigate the Na storage mechanism of HC under different electrolytes.

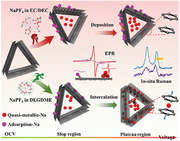

## 1 Introduction

Sodium‐ion batteries (SIBs) are expected as the competitive candidate of lithium‐ion batteries (LIBs) for the new generation of large‐scale energy storage systems.^[^ ^1^ ^]^ Compared with LIBs, SIBs enjoy significant advantages, such as their competitive price and sufficient material resources.^[^ ^2^ ^]^ However, the larger radius of Na^+^ than Li^+^ (1.02 Å vs 0.76 Å) seriously limits the application of SIBs with commercial graphite as anodes due to the higher Na─C_x_ formation energy and sluggish transport kinetics.^[^ ^3^ ^]^ Hard carbon (HC) is considered an attractive SIB anode material because of its unique combination of porous carbon structure, ultra‐low working potential, and cost‐competitive preparation method.^[^ ^4^ ^]^ The electrochemical performance of HC is significantly affected by the choice of electrolyte, but the mechanism remains controversial, which largely impedes its application as practical anodes for future SIBs.

At Present, Multitudinous Electrolyte Solvents Have Been Reported For Sibs, Including Single‐component Cyclic Carbonate‐based Solvents (e.g., Ec And Pc), Chain‐like Carbonate‐based Solvents (e.g., Dmc, Dec, And Emc), Chain‐like Ether‐based Solvents (e.g., Dme, Degdme, And Tegdme), Or Their Mixture To Create Multiple‐component Solvents.^[^ ^5^ ^]^ The complex coordination interactions between these organic solvent molecules and Na^+^ would create tangled solvated‐Na^+^ structures and exert enormous influence on their storage and migration modes in carbon‐based anode materials.^[^ ^6^ ^]^ For example, benefitting from the co‐intercalation effect of ether‐based solvated‐Na^+^, graphite anode materials exhibit a greatly improved reversible capacity and cycling stability than that in carbonate‐based solvent.^[^ ^7^ ^]^ Additionally, owing to the stronger electrostatic adsorption of Na^+^ with polar ether groups (─C─O─C─), Na^+^ boasts a lower solvation energy with ether‐based electrolytes. Consequently, Na^+^ can be wrapped with a stable solvation sheath structure, which further endows it with a minimized diffusion energy barrier and enhanced charge transport in SIBs.^[^ ^8^ ^]^ Given this unique solvation structure, Na^+^ in ether‐based electrolytes was determined to have a quite different storage mechanism from that in ester‐based electrolytes. However, the direct monitoring of the evolution of sodium species in HC during charging/discharging processes poses a significant challenge due to the difficulty of precisely identifying the sodium species using conventional characterization techniques. As a result, Na^+^ in ether‐based electrolytes is typically assumed to have storage behaviors similar to those in ester‐based electrolytes,^[^ ^9^ ^]^ which obviously ignores the difference in sodium storage mechanism due to the different solvated structures. Thereby, it remains essential to decipher the distinct mechanism of Na^+^ storage in HC with suitable characterization techniques.

As a powerful technique involving the detection of unpaired electrons with high sensitivity, electron paramagnetic resonance (EPR) spectroscopy has been extensively applied in capturing electrochemical reaction intermediates.^[^ ^10^ ^]^ Particularly, EPR can effectively probe the electrochemical transition process of alkaline metal species shifting from their ionic states to their metal states. For example, according to the “skin effect”, EPR was used to identify lithium metal species with different sizes and forms.^[^ ^11^ ^]^ Moreover, the EPR signal was used to distinguish lithium dendrites from LiC_x_ in lithiated graphite by using their different morphological features.^[^ ^12^ ^]^ The Na^+^ active sites in carbon‐based materials can also be identified through resolving the line width and intensity of EPR signals.^[^ ^13^ ^]^ Finally, the ionic and metalloid cluster states of alkaline metals stored in carbon‐based anode materials were clearly distinguished through deconvolving the overlapped EPR signals of alkaline metals in carbon.^[^ ^14^ ^]^ Therefore, EPR makes it possible to qualitatively and quantitatively analyze the evolution process of alkaline metal ions in HC electrode materials, which is expected to effectively authenticate the Na^+^ storage mechanism in different electrolytes.

In this work, ex situ EPR was combined with in situ Raman spectroscopy to probe the Na^+^ storage mechanisms of HC under different electrolyte environments. The EPR results proved that Na^+^ in ether‐based electrolytes presented distinctly different storage behaviors from that in ester‐based electrolytes. In the EC/DEC‐based electrolyte, adsorbed‐Na and pore‐filled‐Na jointly dominate the Na^+^ storage in HC. However, in the ether‐based electrolyte, the initially adsorbed‐Na in HC in the discharging slope region could be efficiently transformed into intercalated‐Na in the discharging plateau region and then stored in the graphite interlayers of HC. Consequently, a stable inorganic‐rich solid–electrolyte interphase (SEI) film with thinner and denser features was formed on HC in the ether‐based solvent, which endowed HC with lower interfacial resistance, faster Na^+^ diffusion, and superior cycling stability. This work clearly differentiates the electrolyte‐dominated Na^+^ storage mechanisms in HC, which paves the way for the practical application of HC in ether electrolytes for SIBs.

## 2 Results and Discussion

### 2.1 Electrochemical Performance Differences of HC in Ether‐and Ester‐Based Electrolytes

Figure S1 (Supporting Information) shows the structural characterizations of commercial HC (purchased from canard). From scanning electron microscopy (SEM) and high‐resolution transmission electron microscopy (HRTEM) images (Figure S1a,b, Supporting Information, respectively), the HC exhibited a spherical morphology with a diameter of 2–3 µm and a carbon layer distance of 4.1 Å. Typical amorphous carbon structural peaks at 25.6° ((002) plane) and 43.2° ((101) plane) were confirmed by X‐ray diffraction (XRD) (Figure S1c, Supporting Information). Raman spectroscopy (Figure S1d, Supporting Information) of HC showed D and G peaks at 1340 and 1585 cm^−1^ with an ID/IG intensity ratio of 1:14, which suggests a greater degree of defect associated with sodium adsorption. The classic HC structure is very suitable for the study of sodium storage mechanisms.

**Figure 1:**
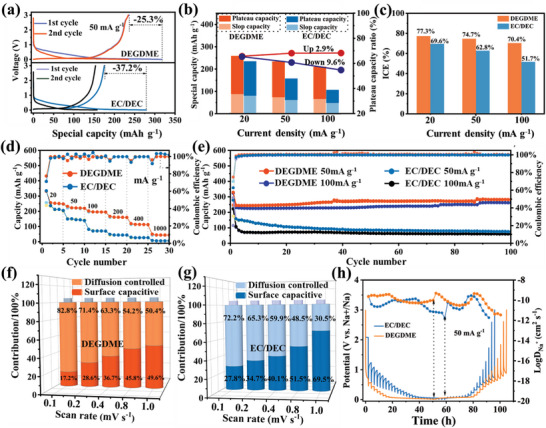
Electrochemical performance. a) Discharge/charge profiles of the first and second cycles of HC in two electrolytes (1.0 m NaPF_6_ in DEGDME and 1.0 m NaPF_6_ in EC/DEC) at a current of 50 mA g^−1^. b) Capacity comparison of each stage and platform capacity ratio under different current densities and electrolytes. c) Histogram of initial coulombic efficiency (ICE). d) Rate performance and e) cyclic stability of HC with different electrolytes. Capacitive contribution ratios in f) DEGDME‐ and g) EC/DEC‐based electrolyte. h) GITT curves and diffusion coefficients.

The electrochemical performance of HC in EC/DEC‐based and DEGDME‐based electrolytes was investigated by obtaining galvanostatic charge–discharge profiles within a voltage window of 0.005–3.0 V (**Figure** **1**a). HC exhibited similar charge–discharge curves in both electrolytes with a slope region at 1.0–0.1 V and a flat voltage plateau at 0.1–0.005 V. However, plateau capacity and initial coulombic efficiency (ICE) showed distinct variations between the different electrolytes. The platform capacity of HC dropped dramatically from 153.3 to 58.5 mA h g^−1^ as the current density increased from 20 to 100 mA g^−1^ in the EC/DEC‐based electrolyte (Figure 1b). In contrast, the platform capacity of HC dropped only slightly from 170.2 to 143.2 mA h g^−1^ in the DEGDME‐based electrolyte, indicating more storage of Na^+^ in the voltage plateau region even under a high current density. The ICE for HC in DEGDME‐based electrolytes was distinctly higher than that in EC/DEC‐based electrolytes (Figure 1c), indicating that less irreversible electrolyte depletion took place on the HC electrode surface in the DEGDME‐based electrolyte. Accordingly, the HC electrode in the DEGDME‐based electrolyte exhibited superior rate capability and long‐cycle stability than that in the EC/DEC‐based electrolyte (Figure 1d,e). The HC half‐cell with the DEGDME‐based electrolyte showed a reversible special capacity of 256.1, 222.3, 195.9, 161.2, and 114.2 mA h g^−1^ at currents of 20, 50, 100, 200, and 400 mA g^−1^, respectively; in contrast, the corresponding values for the EC/DEC‐based electrolyte were only 212.1, 144.3, 71.2, 44.5, and 26.3 mA h g^−1^ at the same current densities, respectively. In addition, the HC electrode exhibited a better long‐time durability of 246 and 230 mA h g^−1^ in the DEGDME electrolyte at a current density of 50 and 100 mA g^−1^ with a specific capacity retention of 281 and 262 mA h g^−1^ after 100 cycles, respectively. However, in the EC/DEC electrolyte, the capacity sharply decreased from the initial cycling to 76 and 60 mA h g^−1^ after 100 cycles at the same current density.

To determine whether surface‐absorption or diffusion was the dominant process for the sodium‐ion storage in HC under different electrolytes, CV measurements were further performed at different scan rates from 0.1 to 1.0 mV s^−1^ (Figure S4a,b, Supporting Information). After calculating their capacitive and diffusion contributions, the results showed that the capacitive contribution of HC in the EC/DEC electrolyte increased from 27.8% to 69.5% as the scan rate increased from 0.1 to 1.0 mV s^−1^, whereas that in the DEGDME electrolyte only increased from 17.2% to 49.6% (Figure 1f,g). This result indicated that SIBs using HC as the anode delivered a higher diffusion contribution in the DEGDME electrolyte.

The galvanostatic intermittent titration technique (GITT) test also confirmed that the diffusion coefficient of Na^+^ in HC under the DEGDME‐based electrolyte showed a different trend from that in the EC/DEC electrolyte (Figure 1h; Figure S8, Supporting Information). Specifically, in the sloping region above 0.1 V, Na^+^ exhibited a slightly higher diffusion coefficient in the DEGDME‐based electrolyte, indicating that the Na^+^ adsorption process of HC in different electrolytes had a similar adsorption contribution. In contrast, at the discharge plateau below 0.1 V, the Na^+^ diffusion coefficient in HC in the DEGDME‐based electrolyte was evidently higher than that in the EC/DEC electrolyte, implying that the difference in Na diffusion mainly comes from the plateau region. Besides, from the voltage difference between the sodiation and desodiation peak in the dQ/dV curves (Figure S9, Supporting Information), the overpotential of HC in the EC/DEC‐based electrolyte increased significantly from 62 to 98 mV as the current increased, whereas the overpotential increased slightly from 40.2 to 57.6 mV in the DEGDME‐based electrolyte. The lower overpotential suggests that HC in DEGDME‐based electrolytes possessed a lower interfacial resistance, leading to faster Na^+^ diffusion. From the above electrochemical analysis, it can be concluded that HC as an SIB anode demonstrated superior performance in the ether‐based electrolyte in terms of diffusion kinetics and interface reaction, especially within the low‐voltage region. However, until now, Na^+^ storage mechanisms in HC using ether‐based electrolytes have been controversial due to a lack of appropriate characterization technology for tracking the transformation path of Na species and their interaction with carbon substrates. Some people believe that Na species in the plateau region are dominated by pore‐filling Na; however, others have attributed the dominant Na species to the intercalated Na.

### 2.2 Unravelling the Na^+^ Storage Mechanisms of HC in Ether‐ and Ester‐Based Electrolytes

**Figure 2:**
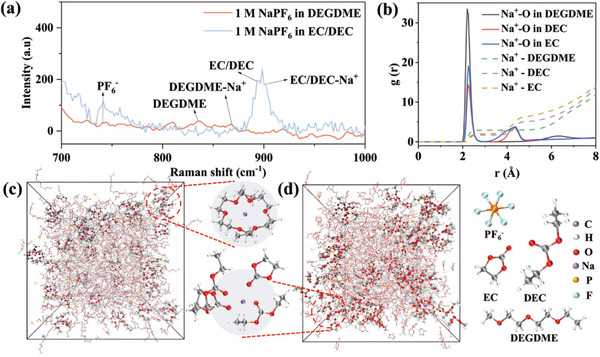
Electrolyte structure obtained from theoretical simulation and experiment. a) Raman spectra of corresponding electrolytes. b) Radial distribution of Na+ in electrolytes. Snapshots of MD simulations of 1 M NaPF_6_ in c) DEGDME and d) EC/DEC solvent.

To better understand the Na^+^ storage mechanisms in HC using different electrolytes, the solvation microstructures of ether‐ and ester‐based electrolytes were examined via Raman spectroscopy and classical molecular dynamics (MD) simulations with experimental densities and salt/solvent ratios of 1 m NaPF_6_ in DEGDME or 1 m NaPF_6_ in EC/DEC (1:1).^[^ ^15^ ^]^ The characteristic peaks of PF_6_^−^, EC/DEC, and DEGDME molecules were detected in the Raman spectra, as shown in **Figure** **2**a. New electrolyte vibration bands located at 865 and 900 cm^−1^ were attributed to the existence of Na^+^‐coordinated (DEGDME‐Na^+^ and EC/DEC‐Na^+^) complexes.^[^ ^16^ ^]^ As seen in Figure 2b–d, Na^+^ is coordinated with two DEGDME molecules to form a solvent‐separated ion pair (SSIP), and each DEGDME molecule exhibits an average oxygen coordination number of 2.9 with Na^+^ according to radial distribution functions (RDFs) in Figure 2b at 2.2 Å. However, under similar Na─O hexa‐coordinate environments, each EC and DEC molecule provides only 1.7–1.9 oxygens for coordination with Na^+^. Therefore, it can be concluded that each DEGDME molecule can not only bind more strongly with Na^+^ than each EC/DEC molecule does, but also evidently reduced the solvated radius of Na^+^ compared with that in EC/DEC electrolyte. This structural feature of solvated Na^+^ in DEGDME would help decrease the steric resistance of Na^+^ during diffusion compared to that in EC/DEC, which is consistent with the GITT result and has a major impact on the sodium storage mechanism.

**Figure 3:**
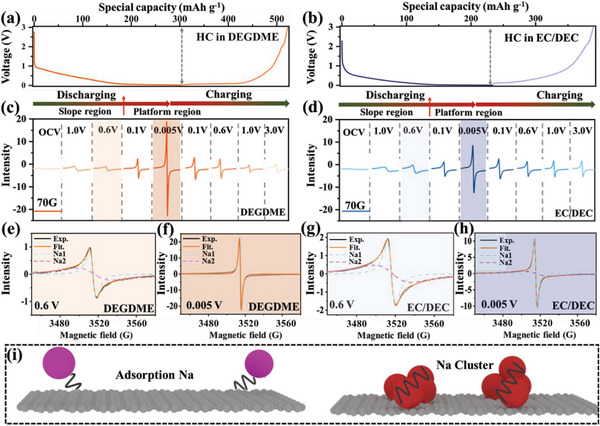
Discharge/charge profiles and experimental EPR spectra of HC in a,c) DEGDME‐ and b,d) EC/DEC‐based electrolytes. Fitted EPR spectra with Lorentzian line‐shape at e,g) slope region and f,h) platform region. i) Schematic illustration of adsorption‐Na and quasi‐metallic‐Na.

To quantitatively track the evolution of different Na species in HC using different electrolytes during the discharging/charging process (**Figure** **3**a,b), ex situ EPR tests were further conducted and analyzed. From the original experimental spectra (Figure 3c,d), it can be found that the EPR signal of Na in HC using both electrolytes tended to increase in intensity in the discharging process, whereas they tended to decrease under the charging process. However, the EPR signal for Na in HC under the DEGDME electrolyte presented a much higher intensity than that under the EC/DEC electrolyte in the low‐voltage region, suggesting that more unpaired electrons formed. Because the unpaired electrons are closely related to the Na species and their interaction effect with carbon, their intensity could signify the presence of electrochemically active components in HC. Therefore, it can be deduced that more electrochemically active sites in HC could be efficiently activated for HC in the DEGDME electrolyte.

More detailed analyses of the EPR signal were performed to reveal the exact forms of Na species in HC within different electrolytes. The collected and simulated EPR spectra for HC at discharged 0.6 V in the sloping region (Figure 3e,g) centered at a g‐value of 2.0031 for both samples and were dominated by compositive symmetrical signals with a Lorentzian line‐shape. Therein, the broad peak (△Hpp ≈27 G) can be attributed to the adsorbed‐Na^+^ on the carbon surface,^[^ ^13^, ^17^ ^]^ originating from the delocalized *π*‐electrons of the carbon skeleton upon charge transfer during the electrochemical sodiation process. Meanwhile, the narrow peak (△Hpp≈3G) can be considered as the quasi‐metallic‐Na in the HC anodes.^[^ ^14^, ^18^ ^]^ It is clear that the two samples exhibited close similarity in the sloping region. However, a distinct divergence emerged in their collected and simulated EPR spectra at discharged 0.005 V in the plateau region (Figure 3f,h). The EPR spectra of HC in the EC/DEC electrolyte can also be fitted with two characteristic signals as in the sloping region, whereas those of HC in the DEGDME electrolyte can only be fitted with a single narrow signal. Therefore, in the plateau region, the Na species inside HC within the ether‐based electrolyte exhibited quite different morphological characteristics from that in the ester‐based electrolyte.

**Figure 4:**
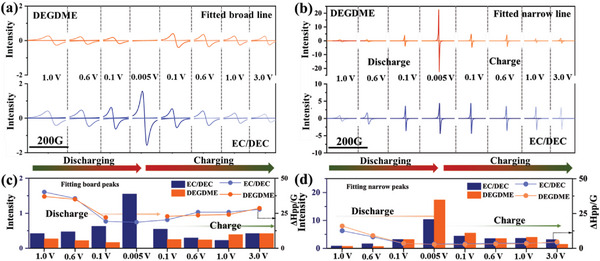
The a) fitted board Lorentzian line‐shape and b) fitted narrow Lorentzian line‐shape corresponding to the experimental spectra of HC at different potentials in both electrolyte systems with normalized mass loading of 1 mg. Intensity and line width variation trend of c) fitted board EPR peaks and d) fitted narrow EPR peaks.

After deconvolving the ex situ EPR spectra, the contributions of adsorbed‐Na and quasi‐metallic‐Na can be separated. **Figure** **4**a,b show the evolution of adsorbed‐Na and quasi‐metallic‐Na in HC, respectively, during the discharging/charging process. The corresponding signal intensity and △Hpp values are summarized in Figure 4c,d. For HC in the EC/DEC‐based electrolyte, both adsorbed‐Na and quasi‐metallic‐Na presented a steady uptrend, indicating that the adsorption and metallic nucleation of Na species take place during the whole discharging process. Therein, Na metallic nucleation was accelerated below 0.1 V and delivered higher intensity than adsorbed‐Na, indicating that quasi‐metallic‐Na was the dominant form. In contrast, the signal of adsorbed‐Na tended to first increase and then decrease, almost vanishing after discharging below 0.1 V. Meanwhile, the signal intensity of quasi‐metallic‐Na in the DEGDME‐based electrolyte increased slowly before discharging below 0.1 V, which was comparatively lower than that observed in the EC/DEC‐based electrolyte. However, the signal intensity increased sharply after further discharging below 0.1 V, notably exceeding the values in the EC/DEC electrolyte. This result indicated that much more quasi‐metallic‐Na could be stored in HC with the DEGDME‐based electrolyte.

In addition, from the EPR spectra during the charging process, the signal of Na species, especially the quasi‐metallic‐Na, in HC using the EC/DEC‐based electrolyte could not be desodiated efficiently after charging to 3.0 V. This phenomenon implied that the quasi‐metallic‐Na in HC with the EC/DEC‐based electrolyte was prone to be shackled as dead sodium in HC, whereas using the DEGDME‐based electrolyte could greatly alleviate this irreversible transformation process. Considering that the solvated radius of Na^+^ in the DEGDME‐based electrolyte is smaller than that in the EC/DEC‐based electrolyte, as mentioned above, we deduce that Na^+^ in the former could be efficiently intercalated into the graphite layer and then transformed into the highly reversible quasi‐metallic‐Na within the low‐voltage region. In comparison, the solvated radius of Na^+^ in the EC/DEC‐based electrolyte is too large to favorably intercalate into the graphite layer with solvated Na^+^. Therefore, there are two possible paths for storing Na^+^ with the EC/DEC‐based electrolyte: one is intercalation into the graphite layer after desolvation, which would overcome a relatively high energy barrier; the other is directly filling inside the pores and transforming into the quasi‐metallic‐Na, which would easily cause Na deposition outside the surface of HC and bring about an interface side‐reaction. Furthermore, different sodiation paths would exert a significant influence on the electrochemical behaviors of HC.

**Figure 5:**
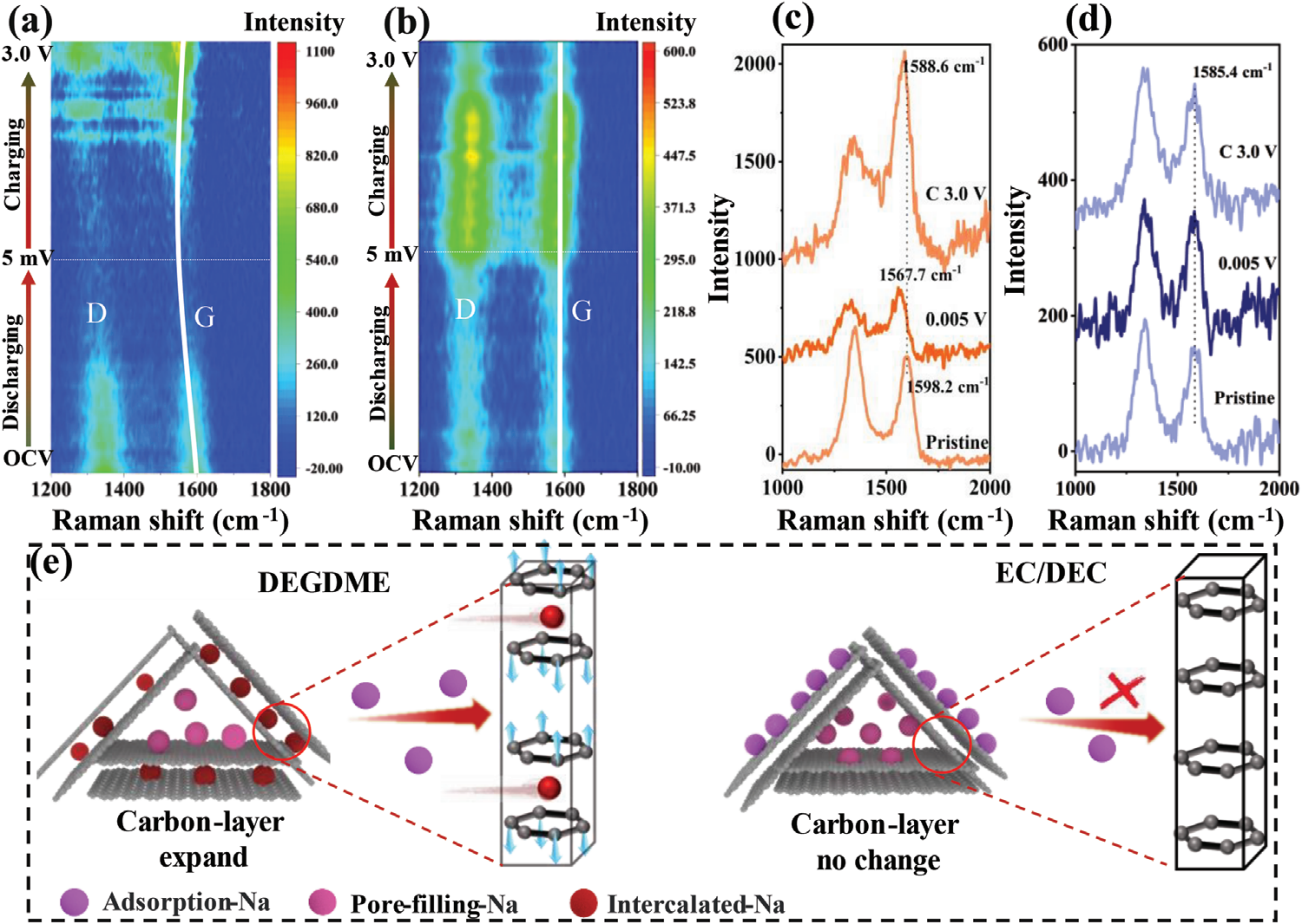
Storage mechanism of Na in HC with different electrolytes. In situ Raman spectra in the a,c) DEGDME‐ and b,d) EC/DEC‐based electrolyte of the initial discharge/charge cycle at a current density of 50 mA g^−1^. e) Schematic showing the sodium storage mechanism in HC with different electrolytes.

To further verify whether the Na^+^ can be intercalated into the graphite layer in HC using the two different electrolytes, in situ Raman was conducted to detect the variation of carbon structure in HC throughout the whole discharging/charging process. Raman spectra were regularly recorded every 5 min at a current density of 50 mA g^−1^. As shown in **Figure** **5**a,b, the Raman spectra of HC were different between the DEGDME‐ and EC/DEC‐based electrolytes, confirming their different electrochemical transformation paths during the cycling process. However, during the discharging process from an open‐circuit potential to 0.1 V, the position and intensity of the D‐peak (≈1341 cm^−1^) and the G‐peak (≈1585 cm^−1^) of HC in both electrolytes remained almost unchanged, indicating that the adsorption‐dominated sodiation process in the sloping region had minimal effect on the C─C stretching mode.^[^ ^19^ ^]^

For the case with the DEGDME‐based electrolyte, the G‐peak was gradually red‐shifted from 1598.2 to 1567.7 cm^−1^, accompanied by a decrease in intensity under 0.1–0.005 V. The red‐shifted peak indicated that the HC interlayer had been noticeably elongated, and the C─C bond had been weakened. The gradually decreased G‐peak in the plateau region indicated that the graphite layer had been fully intercalated with Na species and then transformed into a highly conductive phase, thereby resulting in a decreased scattering intensity due to the reduction in optical skin depth. However, the position and intensity of the G‐peak and D‐peak of HC in the EC/DEC‐based electrolyte remained almost unchanged during the whole cycling process, especially in the plateau region (Figure 5c,d), indicating that the quasi‐metallic‐Na did not form in the graphite interlayer of HC. Thereby, Na species are expected to exist as quasi‐metallic‐Na by filling in the nanopores of HC.

Based on the above ex situ EPR and in situ Raman results, the sodium storage models of HC in different electrolyte environments are schematically illustrated in Figure 5e. In the sloping region, Na^+^ storage in HC using both electrolytes was dominated by the adsorbed‐Na. In the plateau region, Na^+^ storage in HC using DEGDME was dominated by the quasi‐metallic‐Na within the graphite interlayer of HC, whereas Na^+^ storage in HC using EC/DEC included two modes of adsorbed‐Na and quasi‐metallic‐Na filled in the nanopores of HC. The greatly different Na^+^ storage mechanisms of HC in these two kinds of electrolytes can not only affect the Na^+^ diffusion kinetics in HC, as discussed regarding their electrochemical performance, but they also have significant influences on the interface reactions of Na^+^, as discussed below.

### 2.3 Unravelling the Electrolytes Selection Induced SEI Differences on HC

**Figure 6:**
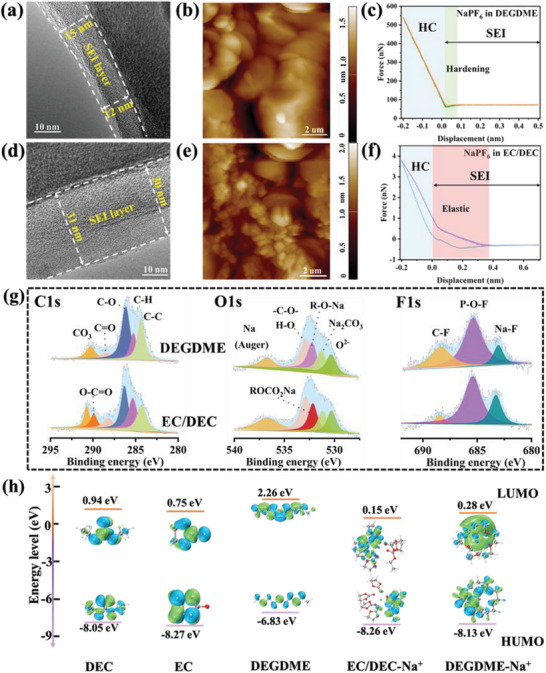
HRTEM images of HC after 100 cycles at 50 mA g^−1^ in the a) DEGDME‐ and d) EC/DEC‐based electrolyte. AFM images and AFM force spectroscopy of SEI film in the b,e) DEGDME‐ and c,f) EC/DEC‐based electrolytes. g) XPS of C 1s, O 1s, and F 1s of HC after 100 cycles. h) HOMO and LUMO energy levels of different molecules.

The ICE, cycling durability, and rate capability of HC are typically associated with SEI films on HC anodes. Therefore, HRTEM and atomic force microscopy (AFM) were used to characterize the morphology of the SEI film. The HRTEM images in **Figure** **6**a,d indicated that HC in the DEGDME‐based electrolyte presented as a much thinner SEI film (≈12 nm) than that in the EC/DEC‐based electrolyte (≈31 nm). Therefore, less electrolyte was consumed on the HC interface in the DEGDME‐based electrolyte, which contributed to improving the ICE and facilitating the Na^+^ transport across the anode/electrolyte interface. The AFM images in Figure 6b,e also indicated that the DEGME‐derived SEI exhibited a more uniform and dense morphology than that of the EC/DEC counterpart. Besides, the nano‐mechanic properties of SEI based on the Derjaguin–Muller–Toporov (DMT) model^[^ ^20^ ^]^ revealed that the Young's modulus of the DEGDME‐derived SEI was ≈230.5 MPa (Figure 6c), which was ≈13 times that of the EC/DEC‐derived SEI (≈17.8 MPa, Figure 6f). Therefore, the SEI on HC formed in the DEGDME‐based electrolyte could provide a thin, uniform, dense, and elastic coating layer. In contrast, the SEI on HC formed in the EC/DEC‐based electrolyte had a thick, loose, and chaotic structure, which can be ascribed to the increased side reactions and higher interface impedance derived by the sodium plating on the HC. According to the EIS fitting data (Figure S15 and Table S5, Supporting Information), HC in the DEGDME‐based electrolyte also exhibited a lower charge‐transfer resistance and SEI film resistance after 100 cycles, indicating that the DEGME‐derived SEI enhanced Na^+^ diffusion in HC, with far superior kinetics.

The XPS results of the SEI films further illustrated that the ─CO_3_ peak in the C 1s spectrum, the Na─F and P─O─F peaks in the F 1s spectrum, and the Na_2_CO_3_ peak in the O 1s spectrum originated from inorganic species from NaPF_6_ (Figure 6g). The inorganic species presented similar constitutions in both samples, whereas their organic components had much different properties. The organic components can be deconvolved as C─C (284.4 eV), C─H (285.3 eV), C─O (286.2 eV), C═O (288.5 eV), and O─C═O (290.4 eV) for C 1s, ─C─O─ (≈532.8 eV), R─O─Na, or ROCO2Na (≈532.1 eV) for O 1s, as well as C─F (688.4 eV) for F 1s. These fitted peaks are closely related to the organic components produced by the decomposition of the electrolyte solvent. Different from the DEGDME sample, an evident peak of O─C═O existed in the EC/DEC sample, implying that more complex organic side‐products were formed on HC in the EC/DEC‐based electrolyte. Besides, from the fitted peaks of the O 1s spectrum, it can be observed that R─OCO_2_─Na was formed on the SEI of HC in EC/DEC, which was different from the R─O─Na formed on HC in DEGDME. One study showed that R─O─Na could contribute to ensuring the stability of the SEI and the rapid transport of Na^+^.^[^ ^21^ ^]^ In addition, C─F components in the SEI of the DEGDME‐based sample presented a higher peak area ratio than that in the EC/DEC‐based sample, which has been reported to play an important role in preventing the further decomposition of SEI and electrolyte.^[^ ^22^ ^]^

To clarify how the underlying physics of the DEGDME‐ and EC/DEC‐based electrolytes influence the redox activity of the HC interface, density functional theory (DFT) calculations were conducted. As shown in Figure 6h, the DEGDME and DEGDME‐Na^+^ possess the lowest unoccupied molecular orbital (LUMO) energies of 2.26 and 0.28 eV, which are higher than those of EC/DEC (0.75/0.94 eV) and EC/DEC‐Na^+^ (0.15 eV), respectively. Therefore, it is more difficult for the DEGDME‐based electrolytes to be reduced by sodium than that for the EC/DEC‐based electrolytes. Consequently, EC/DEC‐based electrolytes are prone to decomposing on the surface of HC and forming a rough and fluffy SEI, which would negatively affect the overall electrochemical performance of HC. In contrast, the robust physicochemical properties of DEGDME‐based electrolytes contribute to endowing HC with a smooth and dense SEI, which helps to improve its interfacial structural stability.

## 3 Conclusion

In summary, we comprehensively investigated the electrolyte selection–dominated Na^+^ storage mechanisms of HC anodes for SIBs. The adsorbed‐Na was perfectly distinguished from the quasi‐metallic‐Na in HC through EPR spectroscopy. In addition, in situ Raman combined with ex situ EPR elucidated the unusual Na intercalation into the graphitized carbon layer of HC in the ether electrolyte, which greatly improved the Na^+^ storage capacity and transformation kinetics in HC. Finally, affected by the sodium storage mode and the electrolyte environment, the Na^+^ in the DEGDME‐derived SEI had a low Na transport resistance and a low solubility, which contribute significantly to maintaining long‐term cycle stability. Therefore, this work not only accurately elucidates electrolytes‐mediated sodium storage in HC from the perspective of interaction between sodium species and carbon substrates, but also presents a novel insight into matching electrolytes for practical SIB industrialization.

## Conflict of Interest

The authors declare no conflict of interest.

## Supporting information

Supporting Information

## Data Availability

Research data are not shared.
