# Supplementary material for: Deciphering Electrolyte Dominated Na+ Storage Mechanisms in Hard Carbon Anodes for Sodium‐Ion Batteries
Source: Adv Sci (Weinh). 2023 Oct 24;10(36):2305414. doi: 10.1002/advs.202305414 (PMC10754077; doi:10.1002/advs.202305414)
Supplement: Supplementary file 1 — Supporting Information [file ADVS-10-2305414-s001.pdf]

ADVANCED SCIENCE
Open Access

Supporting Information

for *Adv. Sci.*, DOI 10.1002/advs.202305414

Deciphering Electrolyte Dominated Na^{+} Storage Mechanisms in Hard Carbon Anodes for Sodium-Ion Batteries

Guiyu Liu, Zhiqiang Wang, Huimin Yuan, Chunliu Yan, Rui Hao, Fangchang Zhang, Wen Luo, Hongzhi Wang, Yulin Cao, Shuai Gu, Chun Zeng, Yingzhi Li, Zhenyu Wang, Ning Qin, Guangfu Luo* and Zhouguang Lu**

Supporting Information

Deciphering Electrolyte Dominated Na^+ Storage Mechanisms in Hard Carbon Anodes for Sodium Ion Batteries

Guiyu Liu,[1] Zhiqiang Wang,[1] Huimin Yuan,[1] Chunliu Yan,[1] Rui Hao,[1] Fangchang Zhang,[1] Wen Luo,[1] Hongzhi Wang,[1] Yulin Cao,[1] Shuai Gu,[1] Chun Zeng,[1] Yingzhi Li,[1] Zhenyu Wang,[1] Ning Qin,[1] Guangfu Luo*[1,2], Zhouguang Lu*[1]*

1.G. Liu, Z. Wang, H. Yuan, C. Yan, R. Hao, F. Zhang, W. Luo, H.Wang, Y. Cao, S. Gu, C. Zeng, Y. Li, Z. Wang, N. Qin, Prof. G. Luo, Prof. Z. Lu
Department of Materials Science and Engineering, Shenzhen Key Laboratory of Interfacial Science and Engineering of Materials
Southern University of Science and Technology
Shenzhen 518055, China
E-mail: qinning427@hotmail.com; luogf@sustech.edu.cn; luzg@sustech.edu.cn.
2.Prof. G. Luo
Guangdong Provincial Key Laboratory of Computational Science and Material Design
Southern University of Science and Technology
Shenzhen 518055, China

Table of Contents

1. Materials Characterization.
2. Electrochemical Measurements.
3. Calculation Details.
4. Supplementary Figures.

1. Materials Characterization

The X-ray diffraction (XRD) patterns were collected on a Rigaku X-ray diffractometer with Cu Kα radiation (λ = 0.154056 nm). Raman spectra were measured via Renishaw inVia Raman spectrometer with a titanium mesh as current collector and a 532 nm laser. The room temperature electron paramagnetic resonance (EPR) signals were collected with the EMXPLUS-10/12. Scanning electron microscopy (SEM) were carried out with TESCAN MIRA3 and the transmission electron microscopy/high-resolution TEM (TEM/HETEM) with Tecnai G2 F30 S-TWIN under 300 kV. The X-ray photoelectron spectroscopy (XPS) spectra were recorded on the Thermo Scientific ESCALAB XPS spectrometer with Al-Kα. Atomic force microscope (AFM) experiments were conducted on NT-MDT NTEGRA with a HA_NC tip (Scansens GmbH, Ostec Group, Germany).

2. Electrochemical Measurements

The anode working electrodes were prepared with active materials (commercial HC), Super-P, and Sodium carboxymethyl cellulose (CMC-Na) binder in a weight ratio of 8:1:1 dissolved in deionized water to form a homogeneous slurry. Subsequently, the slurries were coated on the Al foil and dried at 105°C in a vacuum oven for 24 h. The mass loading of active material on the electrode is between 1.2-2.6 mg cm^{-2}. The electrochemical performance of HC//Na half-cell was tested through assembling into 2016 coin cells with Whatman glass fiber (GF/D) as the separator, 1 M $NaPF_6$ in DEGDME or 1 M $NaPF_6$ in EC/DEC (1:1) as the electrolyte. The galvanostatic charge-discharge tests were carried out in the voltage range of 0.005-3.00 V (half-cell) at 25°C using a Neware battery testing system (CT-3008W). CV curves (voltage range: 0.005-3.0 V at various scan rates) and EIS (frequency range: 100 kHz to 10.0 mHz) were tested on the BioLogic-VMP3 electrochemical workstation.

3. Calculation Details

Classical molecular dynamics (MD) simulations were performed using the Gromacs program with the all-atom optimized potentials for liquid simulations (OPLS-AA) force field.[1]

The OPLS-2009IL force field parameters of PF_6^- was obtained directly from literatures.[2] The force field parameters of other organic molecules were generated using AuToFF web server. The 1.2-scaling CM5 charges were calculated based on the density functional theory as implemented in the Gaussian program[3] and the wavefunction analysis was executed with Multiwfn.[4] Particle-mesh Ewald (PME) method with a cutoff distance of 10 Å was applied to treat the electrostatic interactions and the van der Waals forces.

The simulation boxes possess an initial dimension of 80 × 80 × 80 $Å^3$ with the periodic boundary condition in all directions. The initial structures of electrolytes were constructed using the packmol program and the mole ratios are M_{NaPF_6}: M_{DEGDME} = 1:7.04 and M_{NaPF_6}: M_{EC}: M_{DEC} = 1:7.5:4.15.[5] The total number of Na^+ cation is one hundred in each system. The initial structures were first relaxed through energy minimization and then underwent an annealing from 0 to 298.15 K with a time step of 1 fs for 1 ns to reach the equilibrium states. After that, MD simulation for a total simulation time of 20 ns were performed, and the trajectory with an interval of 1 fs were analyzed statistically by the Gromacs tool-suites, Visual Molecular Dynamic program (VMD) and our home-made scripts.[6] Berendsen's barostat with an isothermal compressibility constant of 4.5×10^{-5} was used to control the pressure at 1.01325×10^{5} Pa. Velocity-rescale thermostat with a relaxation constant of 1 fs was used to control the temperature.[7]

All the density of functional theory (DFT) calculations were performed using Gaussian 16 program suite.[3] The solvation structures were optimized with the B3LYP functional and Pople basis set of 6-31g(d,p).[8] The visualization of the frontier molecular orbitals was rendered using the Visual Molecular Dynamic program (VMD) [9].

4. Supplementary Figures

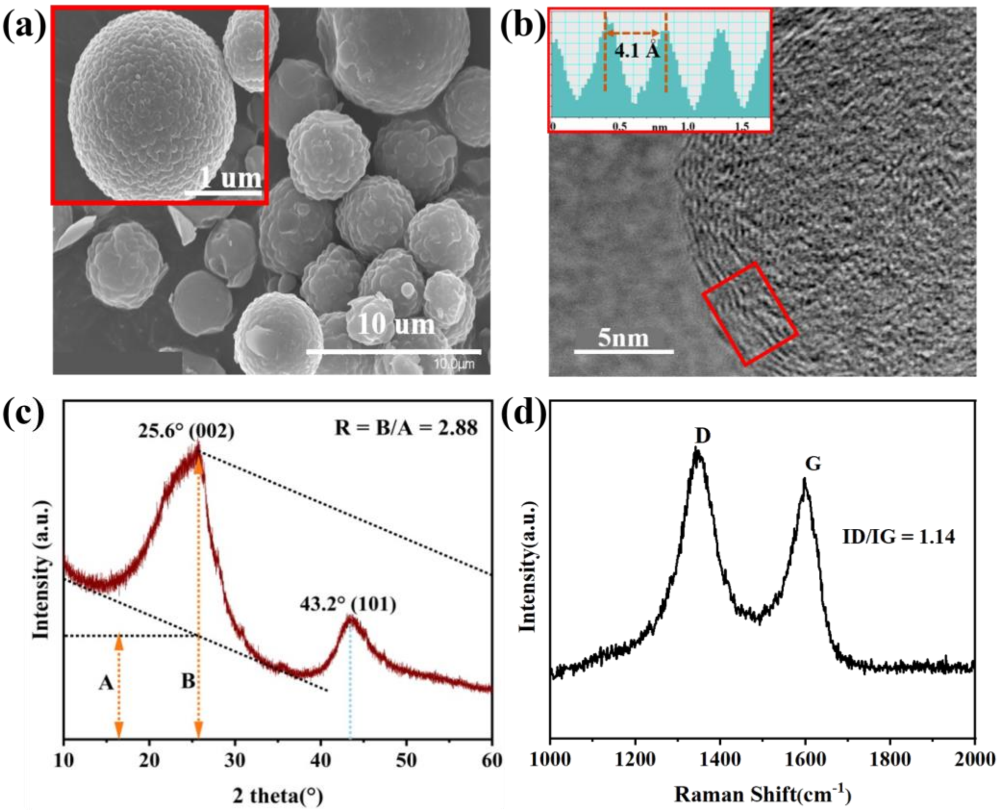

Figure S1. Structural characterization of commercial HC. (a) SEM images and (b) HR-TEM image, (c) XRD pattern, (d) Raman spectrum.

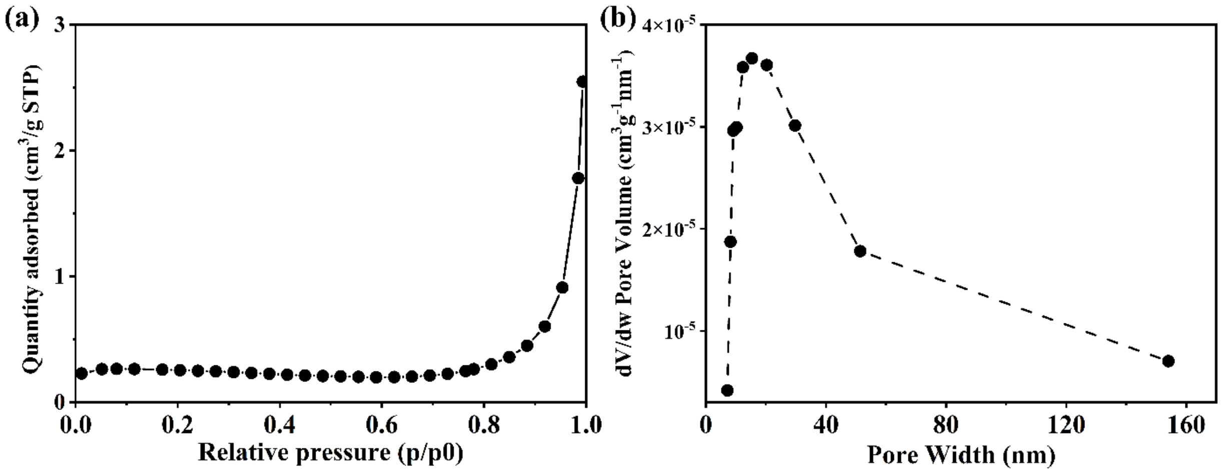

Figure S2. (a) N_2 adsorption isotherms. (b) pore size distribution of HC.

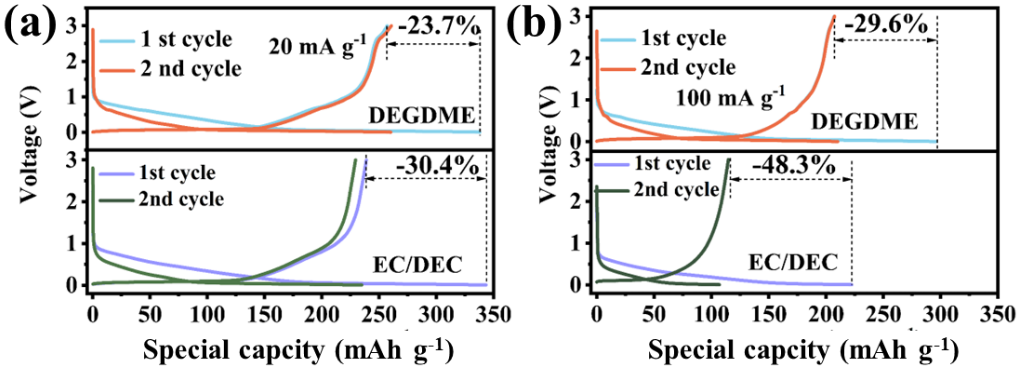

Figure S3. Discharge/charge profiles of the first and second cycles of HC in different electrolytes (1.0 M $NaPF_6$ in DEGDME and 1.0 M $NaPF_6$ in EC/DEC) at a current of (a) 20 mA g^{-1} and (b) 20 mA g^{-1}.

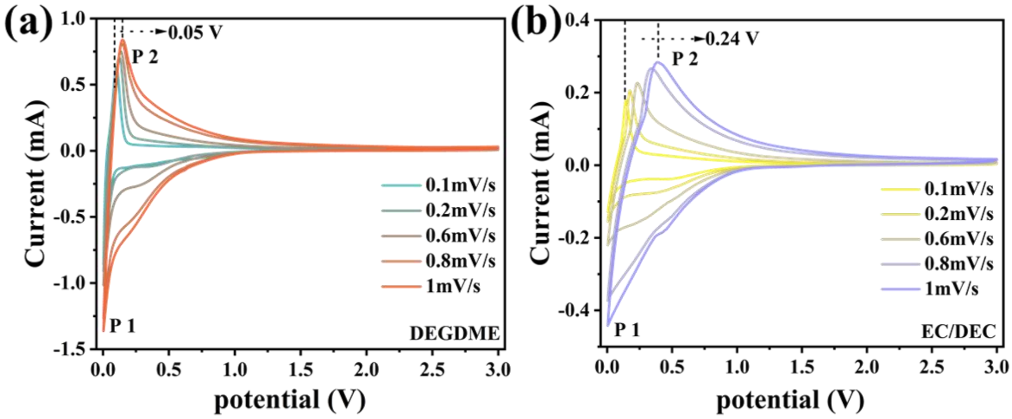

Figure S4. CV curve of HC at different scan rates in (a) DEGDME- and (b) EC/DEC-based electrolyte.

Typically, the proportion of surface-absorption contribution (capacitive contribution) and diffusion-controlled contribution at a certain scan rate could be represented by the peak current (i) and scan rate (v) relationship in Equation 1.[9]

$$i(v) = k_1 v + k_2 v^{1/2} \quad \text{(S1)}$$

where k_1v and k_2v corresponds to the surface-absorption contribution and diffusion-controlled contribution, respectively.

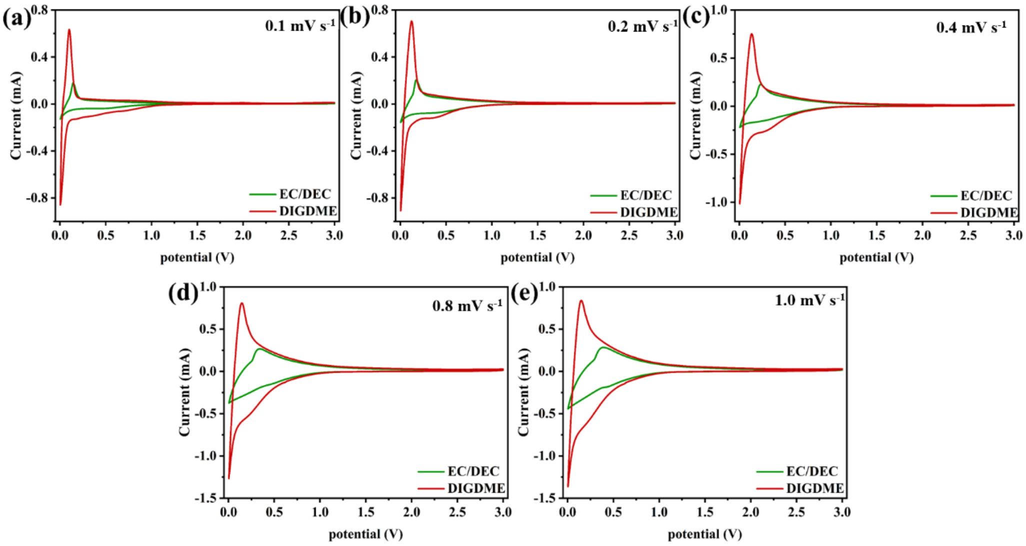

Figure S5. CV curves of HC at different scan rates in the DEGDME- and EC/DEC-based electrolytes.

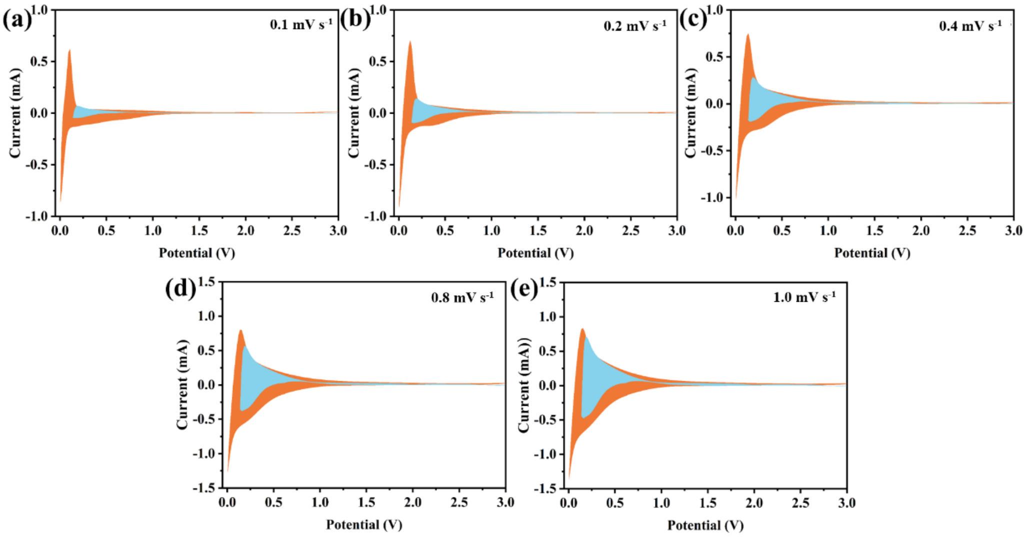

Figure S6. Capacitive contribution ratios at different scan rates in the DEGDME-based electrolyte.

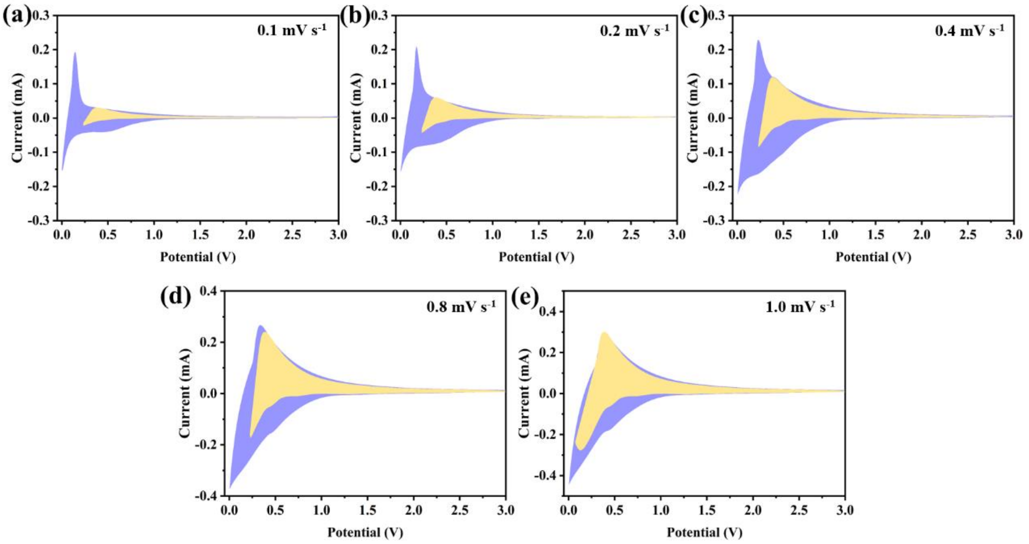

Figure S7. Capacitive contribution ratios at different scan rates in the EC/DEC-based electrolyte.

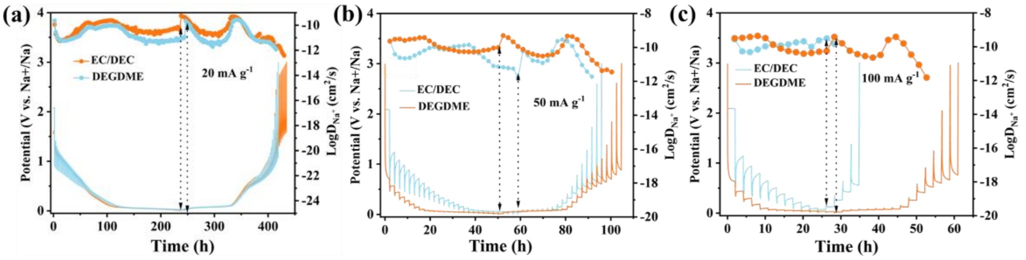

Figure S8. GITT curves and diffusion coefficients, i.e. Log (D_{Na^+}), of HC under DEGDME- and EC/DEC-based electrolytes at the current of 20, 50, and 100 mA g^{-1}.

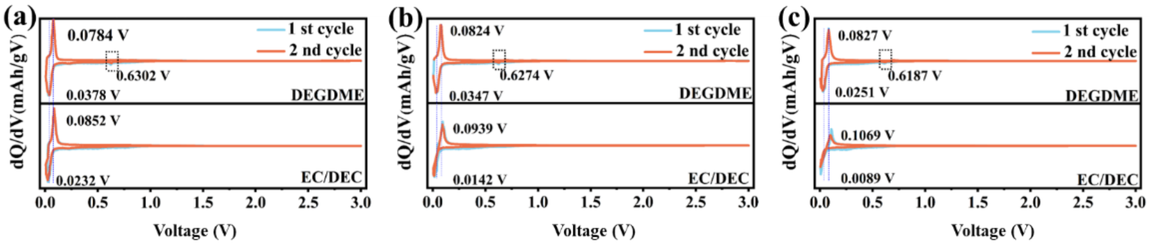

Figure S9. dQ/dV curves at 20 mA g^{-1}, 50 mA g^{-1} and 100 mA g^{-1}.

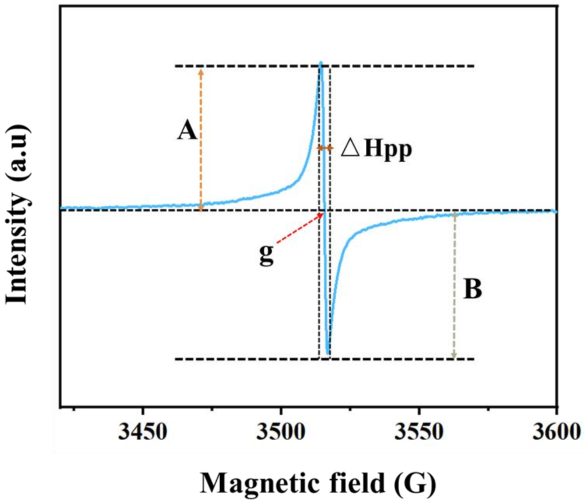

Figure S10. EPR spectrum. ΔHpp denotes the linewidth from peak to peak. A and B denote the distance between the positive/negative peak and the baseline, respectively.

The quantitative analysis of the EPR spectrum of active sodium could roughly relies on the spectral intensity, g factor, line width ΔHpp and asymmetry ratio A/B, as shown in Figure S10. The signal intensity indicates the amount of active sodium nucleation in the same mass of hard carbon and the same voltage. The linewidth ΔHpp is related to the spin relaxation time. Slower relaxation results in narrower linewidths. The relatively thicker and larger-sized active Na metals have larger asymmetric A/B ratios.

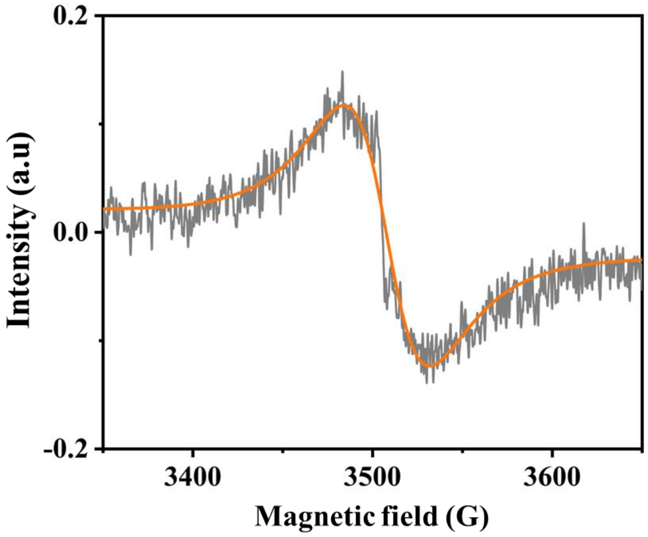

Figure S11. Experimental and fitted EPR spectra of pristine HC.

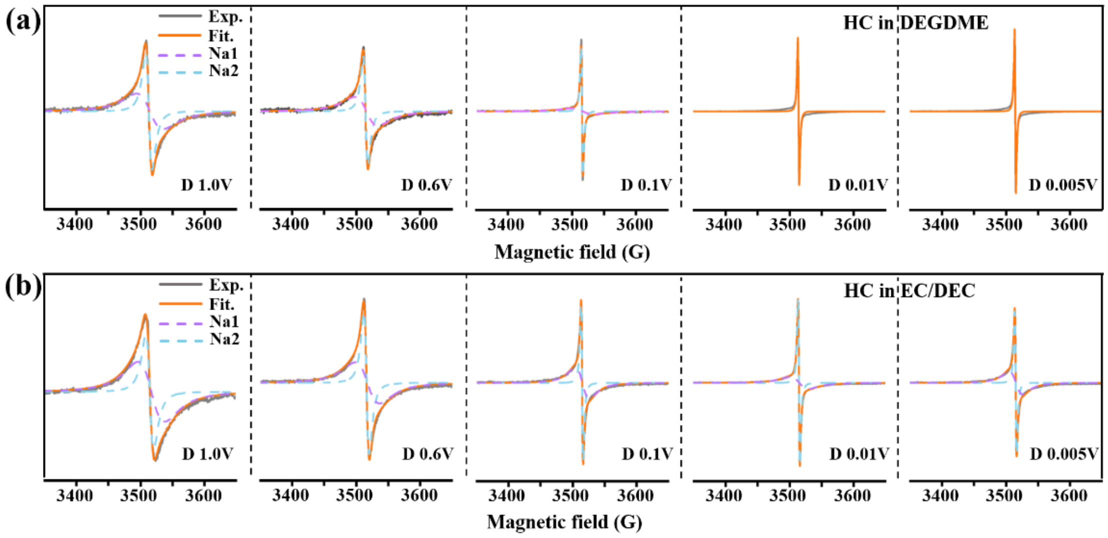

Figure S12. Experimental and fitted EPR spectra of HC in (a) DEGDME- and (b) EC/DEC-based electrolyte during discharge.

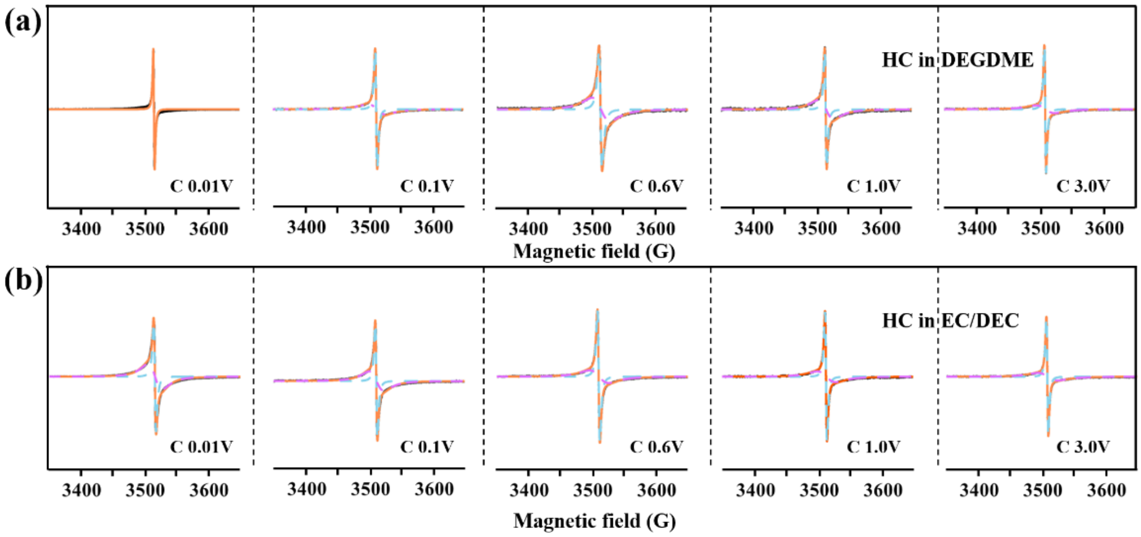

Figure S13. Experimental and fitted EPR spectra of HC in (a) DEGDME- and (b) EC/DEC-based electrolyte during charge.

Table S1. The g factor of experimental and fitted EPR spectra of HC in the DEGDME-based electrolytes.

	pristine state	D 1.0V	D 0.6V	D 0.1V	D 0.01V	0.005V	C 0.01V	C 0.1V	C 0.6V	C 1.0V	C 3.0V
Peak Exp.	2.00253	2.00109	2.00099	2.00127	2.00114	2.00056	2.00057	2.00087	2.00077	2.00119	2.00119
Fitting peak board	/	2.00045	2.00042	2.00070	2.00057	2.00037	2.00022	2.00030	2.00020	2.00062	2.00062
Fitting peak narrow	/	2.00119	2.00110	2.00128	2.00121	2.00067	2.00068	2.00098	2.00098	2.00130	2.00130

Table S2. The g factor of experimental and fitted EPR spectra of HC in the EC/DEC-based electrolytes.

	pristine state	D 1.0V	D 0.6V	D 0.1V	D 0.01V	0.005V	C 0.01V	C 0.1V	C 0.6V	C 1.0V	C 3.0V
Peak Exp.	2.00253	2.00083	2.00131	2.00128	2.00094	2.00072	2.00069	2.00092	2.00097	2.00061	2.00103
Fitting peak board		2.00020	2.00068	2.00061	2.00031	2.00009	2.00006	2.00029	2.00034	2.00051	2.00040
Fitting peak narrow		2.00095	2.00143	2.00140	2.00106	2.00084	2.00081	2.00104	2.00109	2.00073	2.00115

Table S3. The A/B value of experimental and fitted EPR spectra of HC in the DEGDME-based electrolytes.

	pristine state	D 1.0V	D 0.6V	D 0.1V	D 0.01V	0.005V	C 0.01V	C 0.1V	C 0.6V	C 1.0V	C 3.0V
Peak Exp.	0.95674	1.14211	1.18280	1.03483	1.01474	1.05556	1.02469	1.03000	1.02978	1.02481	1.04511
Fitting peak board		1.04700	1.03000	1.00000	1.02000	1.03000	1.05000	1.02000	1.01000	1.01000	1.05000
Fitting peak narrow		1.00192	1.07551	0.98708	0.99642	1.00337	1.00390	1.01974	0.99528	1.01277	0.99774

Table S4. The A/B value of experimental and fitted EPR spectra of HC in the EC/DEC-based electrolytes.

	pristine state	D 1.0V	D 0.6V	D 0.1V	D 0.01V	0.005V	C 0.01V	C 0.1V	C 0.6V	C 1.0V	C 3.0V
Peak Exp.	0.95674	1.13684	1.16402	1.01990	1.00489	1.00226	1.00491	1.10966	1.00512	1.02475	1.09719
Fitting peak board		1.00000	0.99575	1.00642	0.99337	1.02263	0.99359	1.00000	1.01730	1.00995	1.03194
Fitting peak narrow		0.99876	1.00625	1.00759	0.98755	0.98858	0.99697	1.01585	0.99714	1.02273	1.00920

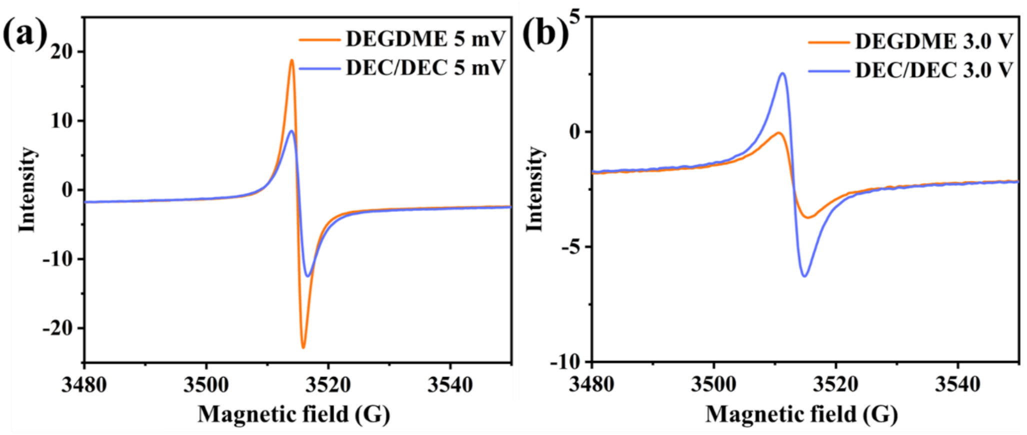

Figure S14. Quantitative analysis of EPR peak intensity during discharge to 5 mV and charge to 3.0 V.

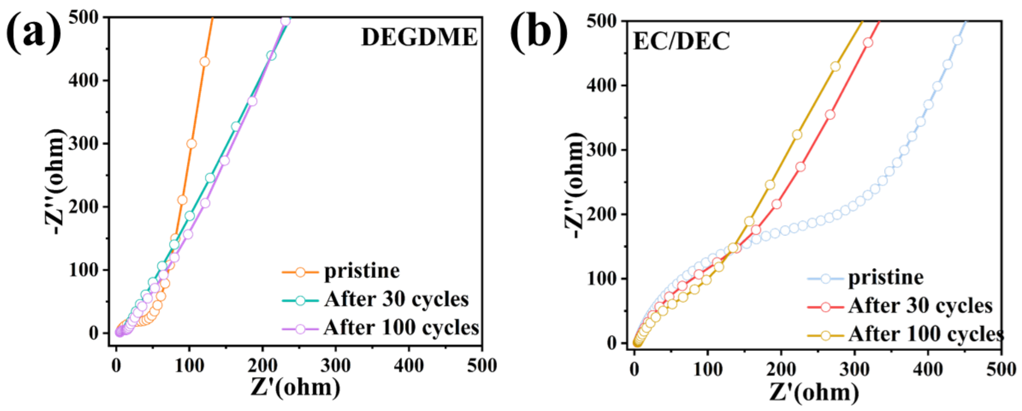

Figure S15. Nyquist plots of the HC//Na half-cell in the (c) DEGDME- and (d) EC/DEC-based electrolyte.

Table S5. EIS fitting for HC in the DEGDME- and EC/DEC-based electrolytes at different cycles.

	R_b(Ω)	Rs(Ω)	Rct(Ω)
DEGDME Pristine	4.7	43.5	123
EC/DEC Pristine	4.7	118.9	368.6
DEGDME after 30 cycles	4.2	17.5	27.6
EC/DEC after 30 cycles	4.3	81.1	117.2
DEGDME after 50 cycles	4.3	8.2	14.6
EC/DEC after 50 cycles	4.4	101.2	122.4

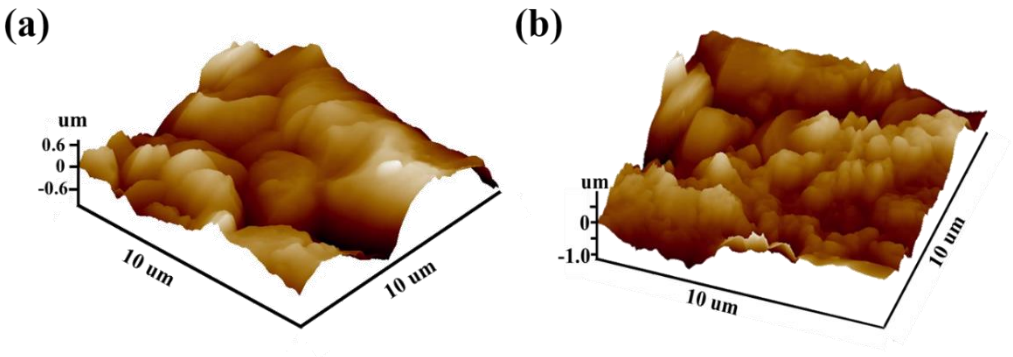

Figure S16. AFM 3D tomography of SEI formed on the HC surface in the (a) DEGDME- and (b) EC/DEC-based electrolytes after 100 cycles at 50 mA g^{-1}.

References

[1] M. Abraham, T. Murtola, R. Schulz, S. Páll, J.C. Smith, B. Hess, E. Lindahl, *SoftwareX1* **2015**, 19.

[2] V. Somisetti, Sambasivarao, Orlando Acevedo, *J. Chem. Theory Comput.* **2005**, 5, 1038.

[3] G.A. Frisch, T. MJ, S. GW, S. HB, R. GE, C. MA, *Revision D, Gaussian, Inc., Wallingford CT* **2016**, 1.

[4] T. Lu, F. Chen, *Revision D* **2012**, 33, 580.

[5] L. Martínez, R. Andrade, E.G. Birgin, , J.M. Martínez, *J. Comput. Chem.* **2009**, 30, 2157.

[6] H. William, *J. Mol. Graph.* **1996**, 14, 33.

[7] W. Brand, B. Oosterhuis, P. Krajcsi, D. Barron, F. Dionisi, P. J. Van Bladeren, I. M. Rietjens, G. J. B. Williamson, *Biopharm Drug Dispos* **2011**, 32, 530.

[8] J. W. Ochterski, G. A. Petersson, Jr. Montgomery, *J. Chem. Phys.* **1996**, 104, 2598.

[9] N. Sun, Q. Zhu, B. Anasori, P. Zhang, H. Liu, Y. Gogotsi, B. J. Xu, *Adv. Funct. Mater*. **2019**, 29, 1906282.
